# The Prognostic Significance of Advanced Lung Cancer Inflammation Index in Patients With Acute Exacerbation of Chronic Obstructive Pulmonary Disease Using Noninvasive Ventilatory Support

**DOI:** 10.1155/carj/2347497

**Published:** 2026-07-03

**Authors:** Sijie Liu, Wei sun, Liming Zhang, Jing Wang

**Affiliations:** ^1^ Department of Respiratory and Critical Care Medicine, Beijing Chao-yang Hospital, Capital Medical University, Beijing, China, ccmu.edu.cn

**Keywords:** acute exacerbation of chronic obstructive pulmonary disease, advanced lung cancer inflammation index, noninvasive ventilatory

## Abstract

**Background:**

The advanced lung cancer inflammation index (ALI) is an important characteristic of cancer and other diseases. This retrospective study aimed to explore the prognostic value of the ALI in patients with acute exacerbation of chronic obstructive pulmonary disease (AECOPD) using noninvasive ventilation (NIV).

**Methods:**

This is a retrospective cohort study. A nonrandom sampling method was employed to collect the inpatients diagnosed with AECOPD and used noninvasive ventilators in the Department of Respiratory and Critical Medicine, Beijing Chao‐yang Hospital, affiliated with Capital Medical University, from January 2017 to December 2022. Participants were categorized into a success group (NIV‐S) and a failure group (NIV‐F) based on the outcome of their noninvasive ventilator use in hospitals. The laboratory indices of both groups were compared. A multivariate logistic regression analysis was conducted to identify the risk factors influencing patient prognosis when using noninvasive ventilators, and the subject working a characteristic curve was employed to assess the predictive power of associated risk factors for the failure of noninvasive ventilators.

**Results:**

A total of 137 patients suffering from AECOPD complicated with respiratory failure were included, including 117 cases in the NIV successful group (NIV‐S, 85.4%) and 20 cases in the NIV failed group (NIV‐F, 14.6%). The arterial blood carbon dioxide partial pressure (PaCO2), fibrinogen, and urea nitrogen in the NIV‐S were lower. At the same time, the total protein, albumin, body mass index (BMI), and ALI were higher than those in the NIV‐F. Moreover, our data revealed that ALI could be considered a reliable biomarker for predicting NIV‐F (AUC: 0.822 and 95% CI: 0.710–0.934). The ALI group, along with an OR of 12.206 (95% CI: 3.086–48.279; *p* < 0.001).

**Conclusions:**

Low ALI is associated with NIV‐F in patients with AECOPD and is a practical and valuable tool for prognostic assessment.

## 1. Introduction

Chronic obstructive pulmonary disease (COPD) is a progressive and highly prevalent respiratory condition characterized by persistent airflow limitation and breathing difficulties. Lung function and quality of life will decrease repeatedly with the acute exacerbation of COPD (AECOPD). On the contrary, medical expenses will increase [1]. Although COPD is incurable, it can be effectively managed through the use of medications, noninvasive ventilation (NIV), and lifestyle modifications. NIV is an effective and safe treatment for managing AECOPD and should be implemented as early as possible in the presence of respiratory acidosis, and the review study found that the use of NIV has been shown to significantly enhance the patient survival rate [2, 3]. As a main treatment of AECOPD, NIV can reduce mortality and tracheal intubation rate, but its comfort is not as good as the high‐flow nasal cannula (HFNC). As we all know, even using NIV in time will likely fail. Some multicenter studies have found that there is no significant difference between nasal high flow and noninvasive respiration in the treatment of mild hypercapnia in AECOPD [4–6]. Therefore, it is necessary to identify the risk of failure in early noninvasive respiratory therapy.

Twenty years ago, researchers focused on the relationship between inflammatory response and nutritional status and combined inflammatory and nutritional indicators to predict the prognosis of tumor patients by considering factors like C‐reactive protein levels and serum albumin (Alb) [7]. The advanced lung cancer inflammation index (ALI) is a new novel index first reported in 2013 by Jafri, a retrospective review, defined as body mass index (BMI) ∗ Alb/neutrophil‐to‐lymphocyte ratio (NLR) [8]. ALI is a crucial prognostic marker for cancer. Several lines of evidence point to the inflammation and nutrient status influencing disease progression in AECOPD. These findings show that Alb and BMI are associated with the prognosis of AECOPD [9–11]^;^ they have a negative correlation. NLR is an independent risk factor affecting prognosis in diseases such as COVID‐19 [12, 13], severe pneumonia [14], and COPD [15, 16], and it may be a factor leading to noninvasive respiratory failure [17]^.^ ALI is a sophisticated metric designed to evaluate inflammation and nutritional status by integrating factors like Alb, BMI, and NLR. There have been many studies on ALI and the prognosis of patients with coronary heart disease and tumors [18, 19].

Therefore, we have reason to suspect that ALI has predictive value for AECOPD using NIV. Currently, no related studies confirm the predictive role of ALI in patients with AECOPD using NIV. The primary objective of this study is to investigate the aforementioned relationship in depth.

## 2. Materials and Methods

### 2.1. Study Design and Population

A total of 137 patients diagnosed with AECOPD and using NIV at Beijing Chao‐Yang Hospital (west campus) from January 2017 to December 2022 were enrolled in this study retrospectively. This retrospective study was approved by our institutional review board (2025‐KE‐344).

Inclusion criteria [17] were age ≥ 18 years, primary diagnosis of COPD determined by spirometry data of airflow obstruction with bronchodilator forced expiratory volume in one second‐to‐forced vital capacity ratio (FEV1/FVC) < 0.7, previous spirometry also considered since a minority of patients had pulmonary function tests during AE period), and admission to hospital due to AECOPD (defined as an acute worsening of respiratory symptoms requiring additional treatment). Indications for NIV use during hospitalization were arterial blood pH < 7.35 and/or arterial blood carbon dioxide partial pressure (PaCO2) > 45 mmHg and/or presence of dyspnea at rest assessed using accessory respiratory muscles or paradoxical abdominal breathing.

Exclusion criteria were age < 18 years, incomplete data, end‐stage chronic diseases, requiring intubation before admission, endotracheal intubation due to other conditions such as heart failure or aspiration, Alb was infused or use of glucocorticoids one week before entering the group, and malignant tumor. Patients with multiple admissions during the study period were selected only for the last visit.

NIV failure (NIV‐F) was defined as requirement of intubation after NIV intervention based on the following criteria: respiratory or cardiac arrest, failure to maintain a ratio of oxygen partial pressure to inspired oxygen concentration (PaO2/FiO2) of > 100, development of conditions necessitating intubation to protect the airway (coma or seizure disorders) or to manage copious tracheal secretions, inability to correct dyspnea, lack of improvement of signs of respiratory muscle fatigue, and hemodynamic instability without response to fluids and vasoactive agents [20, 21].

We have specified that all baseline laboratory data (including ABG, lactate, and fibrinogen) were collected within 48 h prior to NIV initiation. Regarding the settings for breathing and parameters, attending physicians adjust them individually based on the patient’s tolerance.

ALI is defined as BMI ∗ Alb/NLR.

BMI is defined as body weight/the square of height.

The oxygenation index is defined as the partial pressure of arterial oxygen/oxygen concentration.

### 2.2. Statistical Analysis

The chi‐square test, *t*‐test, or nonparametric test was employed to analyze the clinical characteristics. For normally distributed data, the mean and standard deviation (SD) were used. For nonnormally distributed data, either the median with interquartile range or the frequency with percentage was reported. Receiver operating characteristic curve (ROC) was conducted to evaluate the efficacy of ALI in predicting NIV‐F among patients with AECOPD. The significance level was set at *p* < 0.05 for a two‐tailed test. We analyzed the multivariate data using Firth’s penalized likelihood logistic regression. This method is specifically designed for small sample sizes. Besides, we performed internal validation using bootstrapping (1000 resamples) to further confirm the stability of the identified risk factors.

## 3. Results

### 3.1. Patient Demographics and Characteristics

The study involved 137 patients. Table 1 summarizes their characteristics. A total of 117 (85.4%) were classified as NIV‐S, while 20 (14.6%) were classified as NIV‐F. There are no significant differences in terms of age, sex, deep‐vein thrombosis or pulmonary thromboembolism (DVT/PTE), emphysema, hypertension, diabetes, chronic heart disease, cerebrovascular diseases, or ever‐smokers were identified between patients with NIV‐S and NIV‐F.

**TABLE 1:** Baseline characteristics.

	**NIV-S (*N* = 117)**	**NIV-F (*N* = 20)**	**p**

Age (years), median (IQR)	71 (63–79)	77 (65–80)	0.180
Male, sex, *n* (%)	83 (70.9)	16 (80.0)	0.571
DVT/PTE, *n* (%)	6 (5.1)	1 (5.0)	1
Emphysema, *n* (%)	63 (53.8)	16 (80.0)	0.052
Hypertension, *n* (%)	55 (47.0)	7 (35.0)	0.319
Diabetes, *n* (%)	21 (17.9)	2 (10.0)	0.579
Chronic heart disease, *n* (%)	40 (34.2)	8 (40.0)	0.615
Cerebrovascular diseases, *n* (%)	1 (0.9)	0 (0)	1
Ever‐smokers, *n* (%)	60 (51.3)	10 (50.0)	0.916

*Note:* PTE, pulmonary thromboembolism.

Abbreviation: DVT = deep‐vein thrombosis.

We found that compared with the NIV‐S group, the NIV‐F group had higher levels of PaCO2, fibrinogen and urea nitrogen, and lower levels of ALI, BMI, total protein, and Alb (Table 2).

**TABLE 2:** Laboratory parameter comparisons among NIV‐S and NIV‐F.

	**NIV-S (*N* = 117)**	**NIV-F (*N* = 20)**	**p**

Leukocytes (× 109/L), median (IQR)	7.7 (5.9–10.2)	8.8 (5.9–10.0)	0.896
Lymphocytes (× 109/L), median (IQR)	1.0 (0.8–1.5)	1.0 (0.6–1.3)	0.301
CRP (mg/L), median (IQR)	8.0 (5.0–26.0)	9.0 (5.3–49.0)	0.360
Hemoglobin (g/L), median (IQR)	137 (122–151)	135 (119–142)	0.136
Platelets (× 109/L), median (IQR)	203 (156–262)	174 (138–212)	0.050
PaCO2 (mmHg), median (IQR)	60.4 (52.6–68.4)	79.1 (56.8–103.0)	< 0.001
PaO2/FiO2 (mmHg), median (IQR)	252 (211–300)	248 (191–267)	0.405
HCO3‐ (mmol/L), median (IQR)	32 (30–35)	34 (31–39)	0.051
D‐dimer (mg/L), median (IQR)	0.2 (0.1–0.4)	0.3 (0.1–0.6)	0.118
Fibrinogen (g/L), median (IQR)	3.2 (2.7–3.8)	3.8 (3.1–4.6)	0.030
FDP (ug/mL), median (IQR)	2.3 (1.2–4.1)	3.2 (1.6–5.2)	0.442
BNP (*p* g/mL), median (IQR)	500 (145–1592)	1004 (275–2015)	0.138
Uric acid (umol/L), median (IQR)	322 (229–398)	260 (216–355)	0.074
Urea nitrogen (mmol/L),median (IQR)	5.7 (4.2–7.3)	8.2 (5.5–11.2)	0.004
Creatinine (umol/L), median (IQR)	68.4 (56.5–85.5)	68.4 (53.4–87.3)	0.852
Total protein (g/L), median (IQR)	63.0 (58.8–68.1)	59.5 (54.5–64.8)	0.022
ALT (U/L), median (IQR)	15.9 (11.8–23.6)	17.2 (13.4–32.3)	0.206
AST (U/L), median (IQR)	20.0 (16.2–23.3)	22.2 (16.8–32.7)	0.080
Total bilirubin (umol/L),median (IQR)	11.5 (7.6–17.0)	13.0 (7.8–20.4)	0.448
LDH (U/L), median (IQR)	199.0 (163.5–228.0)	212.3 (160.5–262.1)	0.337
Serum iron (umol/L), median (IQR)	9.9 (6.3–14.7)	6.2 (4.3–10.2)	0.063
ALI	18.8 (11.1–28.1)	4.4 (3.8–10.0)	< 0.001
BMI (kg/m2), median (IQR)	21.8 (18.4–25.3)	17.4 (15.0–19.5)	< 0.001
NLR	9.9 (7.8–16.7)	10.8 (7.1–17.4)	0.661
Albumin (g/L), median (IQR)	35.6 (32.3–38.9)	33.0 (29.3–36.8)	0.040

*Note:* PaO2, partial pressure of oxygen; PaCO2, partial pressure of carbon dioxide; HCO3–, bicarbonate; BNP, B‐type natriuretic peptide; ALT, alanine aminotransferase; AST, aspartate aminotransferase; LDH, lactate dehydrogenase; ALI, advanced lung cancer inflammation index; NLR, neutrophil‐to‐lymphocyte ratio. FiO2, fraction of inspired oxygen.

Abbreviations: BMI = body mass index, CRP = C‐reactive protein, FDP = fibrin degradation product, NIV‐F = NIV failure, NIV‐S = NIV success.

### 3.2. Risk Factors of NIV‐F

**FIGURE 1:**
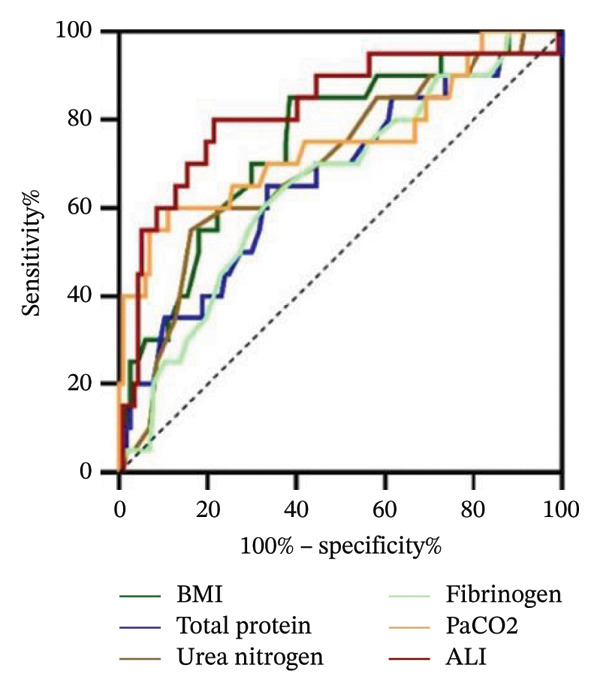
ROC curve of BMI, total protein, urea nitrogen, fibrinogen, PaCO2, and ALI for predicting NIV failure.

The ROC curves for BMI, total protein, urea nitrogen, fibrinogen, PaCO2, and ALI in the NIV‐F group are presented in Figure 1. In Table 3, the AUC for ALI (0.822, 95% CI 0.710–0.934) was higher than those for BMI (0.750, 95% CI 0.636–0.864), total protein (0.661, 95% CI 0.526–0.797), urea nitrogen (0.699, 95% CI 0.573–0.826), fibrinogen (0.653, 95% CI 0.526–0.781), and PaCO2 (0.748, 95% CI 0.610–0.886).

**TABLE 3:** ROC data.

	**Cutoff**	**Sensitivity**	**Specificity**	**Youden’s index**	**AUC**	**95% CI**

BMI	19.9	0.615	0.850	0.465	0.750	0.636–0.864
Total protein	60.9	0.667	0.650	0.317	0.661	0.526–0.797
Urea nitrogen	7.9	0.600	0.829	0.429	0.699	0.573–0.826
Fibrinogen	3.4	0.650	0.650	0.300	0.653	0.526–0.781
PaCO2	76.1	0.600	0.889	0.489	0.748	0.610–0.886
ALI	10.4	0.786	0.800	0.586	0.822	0.710–0.934

	**Positive likelihood ratio**		**Negative likelihood ratio**	**Positive predictive value**		**Negative predictive value**

BMI	4.100		0.453			
Total protein	1.906		0.512			
Urea nitrogen	3.509		0.483			
Fibrinogen	1.857		0.538			
PaCO2	5.405		0.450			
ALI	3.930		0.268	0.390		0.960

*Note:* ALI, advanced lung cancer inflammation index; PaCO2, partial pressure of carbon dioxide.

Abbreviation: BMI = body mass index.

**FIGURE 2:**
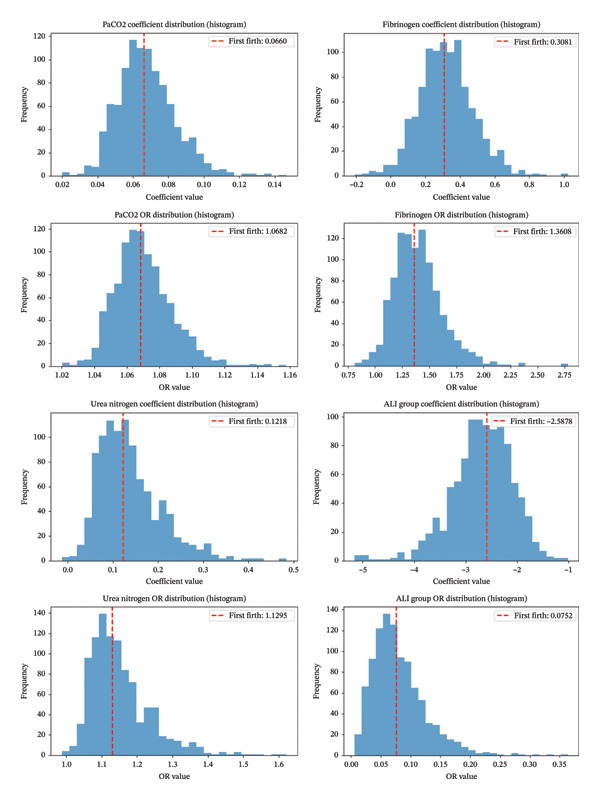
Bootstrapping (1000 resamples).

The cutoff for predicting NIV‐F was ALI > 10.4; the population was divided into a high ALI group (H‐ALI) and a low ALI group (L‐ALI), and the data differences were analyzed between the two groups in Table 4. The two groups significantly differed in lymphocyte, hemoglobin, HCO3‐, fibrinogen, BNP, total protein, Alb, serum iron, BMI, and NLR. Associations between NIV‐F and clinical parameters were analyzed by multivariate logistic regression, which showed that the ALI group (OR: 12.206 and 95% CI: 3.086–48.279) was independently associated with NIV‐F (Table 5). We reanalyzed the multivariate data using Firth’s penalized likelihood logistic regression. This method is specifically designed for small sample sizes. Besides, we performed internal validation using bootstrapping (1000 resamples) to further confirm the stability of the identified risk factors (Table 6 and Figure 2).

**TABLE 4:** Laboratory‐parameter comparisons among L‐ALI and H‐ALI.

	**Total (*N* = 137)**	**L-ALI (*N* = 41)**	**H-ALI (*N* = 96)**	**p**

Leukocytes (× 109/L), median (IQR)	7.7 (5.9–10.2)	7.7 (6.1–10.3)	7.8 (5.6–10.1)	0.655
Lymphocytes (× 109/L), median (IQR)	1.0 (0.8–1.4)	1.0 (0.7–1.2)	1.1 (0.9–1.6)	0.022
CRP (mg/L), median (IQR)	8.0 (5.0–27.0)	10.0 (5.0–73.0)	8.0 (5.0–25.5)	0.218
Hemoglobin (g/L), median (IQR)	136 (122–149)	128 (118–144)	138 (123–152)	0.022
Platelets (× 109/L), median (IQR)	196 (153–255)	205 (149–288)	192 (155–249)	0.427
PaCO2 (mmHg), median (IQR)	61.3 (53.0–73.0)	65.0 (53.3–79.1)	60.3 (53.0–68.6)	0.081
PaO2/FiO2 (mmHg), median (IQR)	251 (208–298)	234 (192–269)	257 (214–307)	0.056
HCO3‐(mmol/L), median (IQR)	33 (30–36)	34 (31–38)	32 (29–35)	0.036
D‐dimer (mg/L), median (IQR)	0.2 (0.1–0.4)	0.2 (0.1–0.5)	0.2 (0.1–0.4)	0.063
Fibrinogen (g/L), median (IQR)	3.3 (2.7–4.0)	3.5 (3.1–4.5)	3.2 (2.6–3.7)	0.002
FDP (ug/mL), median (IQR)	2.4 (1.2–4.2)	3.2 (1.4–5.7)	2.3 (1.1–3.8)	0.059
BNP (*p* g/mL), median (IQR)	500 (172–1617)	1153 (172–2208)	424 (135–946)	0.004
Uric acid (umol/L), median (IQR)	317 (225–388)	277 (210–370)	322 (235–397)	0.183
Urea nitrogen (mmol/L), median (IQR)	6.0 (4.3–7.8)	6.5 (4.6–9.6)	5.7 (4.2–7.4)	0.106
Creatinine (umol/L), median (IQR)	68.4 (56.3–85.5)	66.1 (52.3–85.3)	69.8 (57.0–85.6)	0.392
Total protein (g/L), median (IQR)	63.0 (58.0–67.6)	59.0 (56.1–62.7)	64.8 (60.1–69.3)	< 0.001
ALT (U/L), median (IQR)	15.9 (12.0–24.1)	17.3 (11.0–26.2)	15.8 (12.0–23.6)	0.570
AST (U/L), median (IQR)	20.1 (16.2–24.2)	21.0 (15.9–28.1)	20.0 (16.4–23.9)	0.972
Total bilirubin (umol/L), median (IQR)	11.6 (7.6–17.0)	11.5 (7.6–15.7)	12.0 (7.7–17.3)	0.726
LDH (U/L), median (IQR)	200.9 (163.0–231.5)	215.4 (160.9–252.0)	193.9 (167.4–226.5)	0.179
Serum iron (umol/L), median (IQR)	8.7 (6.1–14.4)	6.3 (4.3–10.8)	10.3 (6.9–15.7)	0.001
BMI (kg/m2), median (IQR)	20.8 (17.7–24.5)	17.3 (16.0–19.4)	22.6 (19.1–25.6)	< 0.001
NLR	10.1 (7.8–14.9)	13.4 (8.8–17.3)	9.7 (7.7–12.8)	0.030
Albumin (g/L), median (IQR)	35.0 (31.9–38.1)	32.4 (28.8–34.8)	36.7 (33.1–39.1)	< 0.001

*Note:* PaO2, partial pressure of oxygen; PaCO2, partial pressure of carbon dioxide; FiO2, fraction of inspired oxygen; HCO3–, bicarbonate; LDH, lactate dehydrogenase; ALT, alanine aminotransferase; AST, aspartate aminotransferase; NLR, neutrophil‐to‐lymphocyte ratio.

Abbreviations: BMI = body mass index, BNP = B‐type natriuretic peptide, CRP = C‐reactive protein, FDP = fibrin degradation product.

**TABLE 5:** ORs for NIV failure (multivariate analysis).

Characteristic	Multivariate analysis		
	*p*	OR	95% CI
PaCO2	< 0.001	0.933	0.898–0.969
Fibrinogen	0.168	0.731	0.469–1.141
Urea nitrogen	0.114	0.890	0.770–1.029
ALI group	< 0.001	12.206	3.086–48.279

*Note:* PaCO2, partial pressure of carbon dioxide.

**TABLE 6:** ORs for NIV failure (Firth’s penalized likelihood logistic regression).

Characteristic	Firth’s penalized likelihood logistic regression		
	*p*	OR	95% CI
PaCO2	< 0.001	1.068	1.028–1.105
Fibrinogen	0.172	1.347	0.879–2.065
Urea nitrogen	0.093	1.124	0.981–1.289
ALI group	< 0.001	0.102	0.029–0.365

*Note:* PaCO2, partial pressure of carbon dioxide.

## 4. Discussion

This study shows that ALI, a combination of nutritional state and inflammation in clinical settings, can predict the clinical benefit of NIV in AECOPD. Patients who scored high on the ALI scale experienced significantly greater success with NIV compared with those with lower ALI scores.

The results of this study indicate that when using ALI as a predictive threshold, the positive predictive value is 0.39, while the negative predictive value is as high as 0.96. This suggests that its clinical application has a clear focus: ruling out NIV‐F. The negative predictive value, close to 1.0, means that when the indicator suggests NIV treatment may be successful, its predictive accuracy is extremely high, with a true success probability of up to 96%. This makes the threshold a reliable indicator for excluding NIV‐F, helping clinicians identify patients suitable for continued NIV and reducing premature or unnecessary tracheal intubation. However, the positive predictive value of only 0.39 indicates low predictive accuracy and a high false positive rate when the indicator suggests NIV may fail. Therefore, this indicator should not be used alone as the basis for initiating invasive ventilation. In clinical practice, the comprehensive evaluation of factors such as the patient’s level of consciousness, respiratory mechanics, and blood gas changes remains necessary to improve decision‐making accuracy. In summary, this indicator holds high value in ruling out NIV‐F in treatment decisions and is more suited for safely screening populations who can continue NIV, rather than serving as a confirmatory indicator for predicting failure.

Growing evidence shows that both inflammatory and nutritional markers predict many diseases, such as cancer [22], cardiovascular disease, and cerebrovascular disease [23]. BMI and inflammatory factors have also some research on the prognosis of AECOPD. Alb, BMI, and NLR express patients’ nutritional status and inflammatory degree. Prior research has shown that AECOPD patients with high Alb, BMI, and low NLR have a better prognosis. ALI is a combination of inflammatory and nutritional indicators to predict the prognosis of tumor patients [24, 25]. In the later stages, ALI was gradually applied to all kinds of diseases, but there was no related research in patients with AECOPD using NIV. The results of our previous study showed that patients with high fibrinogen had a high probability of failure in patients who have AECOPD and are using NIV. Still, the nutritional status of the patients was not comprehensively analyzed [17].

A study showed that the inflammatory response varies between infection‐induced AECOPD and uninfected AECOPD [26]. The main triggering factor of AECOPD is viral infection, followed by bacterial infection. During viral infection, lymphocytes decrease, while leukocytes and neutrophils increase in response to bacterial infection, increasing NLR. At the same time, as inflammatory factors, neutrophils can secrete various cytokines to regulate the function of epithelial cells, mast cells, and macrophages and contribute to the inflammatory response. In contrast, lymphocytes play a role in immune regulation and immune response. The imbalance between the two will make the progress of AECOPD difficult to control; Lee et al. discovered that the NLR is linked to the severity of airflow limitation and can serve as a predictor for the failure of NIV [27].

Independent risk factors for death in COPD patients include low BMI (HR: 0.922 and 95% CI: 0.883–0.963), as well as increased age and decreased FEV1 [28]. Hallin found that patients with a BMI < 20 kg/m^2^ had a higher risk of death than those with a higher BMI [29]^.^ Low MI indicates poor nutritional status of the patient, leading to insufficient strength of respiratory muscles, poor exercise endurance, and impaired pulmonary respiratory function, which in turn affects the therapeutic effect of NIV. Alb serves as an independent risk factor for the death of AECOPD in hospitals, which is negatively correlated [16]. The decrease of Alb and the imbalance of albumin/globulin ratio (AGR) have been confirmed to serve as a reference index for assessing the condition of elderly patients with AECOPD, identifying combined infections, and predicting the prognosis [30]. Low Alb levels and weak respiratory muscles affect lung function and the therapeutic effect of NIV. Pacilli [31] found that the most significant risk factor for the failure of NIV treatment in AECOPD complicated with respiratory failure is the serum Alb level, which is consistent with the findings of this study.Factors affecting the failure of NIV include higher PaCO_2_ and fibrinogen. In this study, the PaCO_2_ is still an independent risk factor for NIV‐F. Still, fibrinogen is inconsistent with other studies, which may be related to the small number of cases in the failure group.

To sum up, nutrition and inflammatory index are the risk factors of NIV‐F in AECOPD. ALI is a combination of the two forms, which is more persuasive. As per our research results, the HR and AUC of the ALI group are higher than other indicators, which is still convincing in the small sample study.

### 4.1. Limitations

Our study has several limitations. First, it was conducted at a single research center, the sample size of 137 patients was relatively small, and the number of patients with NIV‐F was particularly low. To rigorously address this statistical limitation without altering the study cohort, we reanalyzed the multivariate data using Firth’s penalized likelihood logistic regression. This method is specifically designed for small sample sizes. Besides, we performed internal validation using bootstrapping (1000 resamples) to further confirm the stability of the identified risk factors. The above results indicate the stability of this sample. Additionally, retrospective design may introduce selection bias and restrict the ability to determine causality.

## 5. Conclusion

ALI score is a crucial predictor of survival in AECOPD using NIV, as in lung cancer. NIV‐F is more common in patients with a low ALI score. The assessment of ALI can identify patients in AECOPD at high risk of NIV‐F and can be a useful prognostic marker in clinical practice. Future research should involve large, well‐designed studies to validate the predictive capabilities of ALI.

## Funding

No funding was received.

## Ethics Statement

The Ethics Committee of Beijing Chao‐yang Hospital (2025‐KE‐344) approved this study by the Declaration of Helsinki.

## Consent

Our study was performed using anonymized healthcare data. Informed consent from each patient was waived.

## Conflicts of Interest

The authors declare no conflicts of interest.

## Data Availability

Due to ethical restrictions, the raw data cannot be made publicly available. However, deidentified data may be obtained from the corresponding author upon reasonable request.
